# Neonatal and Maternal Complications of Placenta Praevia and Its Risk Factors in Tikur Anbessa Specialized and Gandhi Memorial Hospitals: Unmatched Case-Control Study

**DOI:** 10.1155/2020/5630296

**Published:** 2020-01-06

**Authors:** Ashete Adere, Abay Mulu, Fikremelekot Temesgen

**Affiliations:** ^1^Woldia University, Ethiopia; ^2^Department of Anatomy, Addis Ababa University, Ethiopia; ^3^Department of Obstetrics and Gynecology, Addis Ababa University, Ethiopia

## Abstract

**Background:**

Placenta praevia is a disorder that happens during pregnancy when the placenta is abnormally placed in the lower uterine segment, which at times covers the cervix. The incidence of placenta praevia is 3-5 per 1000 pregnancies worldwide and is still rising because of increasing caesarean section rates.

**Objective:**

To assess and identify the risk factors and maternal and neonatal complications associated with placenta praevia. *Method and Materials*. Target populations for this study were all women diagnosed with placenta praevia transvaginally or transabdominally either during the second and third trimesters of pregnancy or intraoperatively in Tikur Anbessa Specialized and Gandhi Memorial Hospitals. The study design was unmatched case-control study. Data was carefully extracted from medical records, reviewed, and analyzed. Unconditional logistic regression analysis was performed using adjusted odds ratios (AOR) with 95% confidence intervals.

**Results:**

Pregnancies complicated by placenta praevia were 303. Six neonatal deaths were recorded in this study. The magnitude of placenta praevia observed was 0.7%. Advanced maternal age (≥35) (AOR 6.3; 95% CI: 3.20, 12.51), multiparity (AOR 2.2; 95% CI: 1.46, 3.46), and previous history of caesarean section (AOR 2.7; 95% CI: 1.64, 4.58) had an increased odds of placenta praevia. Postpartum anemia (AOR 14.6; 95% CI: 6.48, 32.87) and blood transfusion 1-3 units (AOR 2.7; 95% CI: 1.10, 6.53) were major maternal complications associated with placenta praevia. Neonates born to women with placenta praevia were at increased risk of respiratory syndrome (AOR 4; 95% CI: 1.24, 13.85), IUGR (AOR 6.3; 95% CI: 1.79, 22.38), and preterm birth (AOR 8; 95% CI: 4.91, 12.90).

**Conclusion:**

Advanced maternal age, multiparity, and previous histories of caesarean section were significantly associated risk factors of placenta praevia. Adverse maternal outcomes associated with placenta praevia were postpartum anemia and the need for blood transfusion. Neonates born from placenta praevia women were also at risk of being born preterm, intrauterine growth restriction, and respiratory distress syndrome.

## 1. Introduction

Placenta praevia is a disorder that happens during pregnancy that is characterized by the presence of placental stissue close to or covering the cervix. The greatest risk of placenta praevia is bleeding. Bleeding often occurs as the lower part of the uterus begins to stretch and lengthen in preparation for delivery. When the cervix begins to efface and dilate, the attachment of the placenta to the uterine wall is detached, resulting in bleeding [1].

All placentas overlying the os (to any degree) are termed praevia, and those near to but not overlying the os are termed low-lying [2].

The incidence of placenta praevia is 3-5 per 1000 pregnancies worldwide, and it is still rising because of increasing caesarean section rates. This is because a uterine scar in the lower segment may attract a low implantation of the placenta. The incidence is much higher at midpregnancy than at 36 weeks and above because of formation of the lower segment of the uterus and possibly due to trophotropism resulting in resolution of placenta praevia [3].

Several studies attempted to define risk factors for placenta praevia, and pointed out an association with advanced maternal age, parity, maternal smoking, infertility treatments, previous caesarean deliveries, previous placenta praevia, and recurrent abortions. Among the aforementioned risk factors, several have increased during the past decades including the rate of caesarean sections, advanced maternal age, and the number of women undergoing infertility treatments [4].

Neonates born to mothers with placenta praevia more likely suffer from preterm birth, perinatal death, congenital malformations, and Apgar scores at 1 minute and 5 minutes lower than 7 [5–11]. Perinatal morbidity is also studied that majority of babies require resuscitation and NICU admission [9]. Moreover, the most substantial outcome of this disorder is small for gestational age and low birth weight [11, 12].

The complication of placenta praevia is limited not only to the antepartum period but also to the intrapartum and postpartum courses which can also be complicated with a high rate of caesarean delivery, peripartum hysterectomy, morbid adherence of placenta, and postpartum hemorrhage [6, 13–16]. Previous studies have estimated the rate of hysterectomy among women with placenta praevia to be 5%. Pregnancies complicated with placenta praevia have also a significantly higher rate of postpartum anemia (OR 5.5, 95% CI: 4.4–6.9) and delayed discharge from the hospital [8, 15].

Studies have shown that placenta praevia also carries greater risks of surgical complications [15]. Therefore, the purpose of the present study was to determine the magnitude, risk factors, and neonatal and maternal outcomes of pregnancies complicated with placenta praevia.

## 2. Materials and Methods

### 2.1. Study Area and Period

The study was conducted in Addis Ababa, capital city of Ethiopia. The city lies at an altitude of 7546 feet (2300 metres). Tikur Anbessa Specialized Referral Hospital and Gandhi Memorial Hospital were selected for this study purposely based on the patient load. The data was collected from March 1 to July 30, 2018.

### 2.2. Study Design

The study design was unmatched case-control study.

### 2.3. Source and Study Population

The source population of this study was the woman medical records in Tikur Anbessa Specialized Referral and Gandhi Memorial Hospitals from September 2015 to January 2018, whereas the study population was all the delivery medical records with singleton pregnancies complicated with placenta praevia at Tikur Anbessa Specialized Referral and Gandhi Memorial Hospitals from September 2015 to January 2018.

### 2.4. Sample Size and Sampling Procedure

All singleton deliveries with placenta praevia that took place at Tikur Anbessa Specialized Referral and Gandhi Memorial Hospital from September 2015 to January 2018 were selected for the study. First, all cases were identified from HMIS (Health Management Information System), and their medical registration number was used to access patient's information. Complete birth registry records were considered for analysis.

From 44342 total deliveries, a total of 303 placenta praevia cases were considered for analysis. Regarding control selection, controls were selected after proportional allocation to each year's total number of deliveries in both hospitals. A systematic random sampling method using the patient's medical registration number was used, and finally, 303 controls without placenta praevia were regarded for the study.

### 2.5. Inclusion and Exclusion Criteria

#### 2.5.1. Inclusion Criteria

*(1) For cases*

- All singleton pregnancies diagnosed with placenta praevia transvaginally or transabdominally either during the second and third trimesters of pregnancy or intraoperatively

*(2) For controls*

- All singleton pregnancies with no diagnosis of placenta praevia

#### 2.5.2. Exclusion Criteria for Both Case and Control

(ii) Women with missing medical notes
(iii) Women with multiple gestation pregnancies also excluded to avoid overrepresentation of studying high-risk women

### 2.6. Study Variables

#### 2.6.1. Independent Variables

- Sociodemographic factors
- Obstetric factors
- Neonatal and maternal complications

#### 2.6.2. Dependent Variables

- Placenta praevia

### 2.7. Operational Definitions

*Postpartum hemorrhage (PPH)*: defined as a blood loss of 500 ml or more within 24 hours after birth.

*Moderate anemia*: corresponds to a hemoglobin level of 7.0-9.9 g/dl.

*Severe anemia*: corresponds to a hemoglobin level of less than 7 g/dl.

*Urinary tract infection (UTI)*: an infection involving any part of the urinary system, including the urethra, bladder, ureters, and kidney.

*Intrauterine growth restriction (IUGR)*: refers to the poor growth of a baby while in the mother's womb during pregnancy.

*Respiratory distress syndrome (RDS)*: the most common lung disease in premature infants and occurs because the baby's lungs are not fully developed.

*Neonatal jaundice*: a yellowish discoloration of the white part of the eyes and skin in a newborn baby due to high bilirubin levels.

*Low birth weight (LBW)*: defined as a birth weight of less than 2500 g (up to and including 2499 g), as per the World Health Organization.

### 2.8. Data Collection Tools

A checklist was designed to collect data about study participant's sociodemographic characteristics, obstetric and gynecological history, history of current pregnancy, mode of delivery, and maternal and neonatal complications.

### 2.9. Data Quality Management

Data was checked for completeness and consistency before data entry by the principal investigator; the completed questionnaire was coded. For data cleaning, the coded data was entered into EPI Info version 3.5.

### 2.10. Data Analysis and Processing

Data was entered into EPI Info version 3.5.1 for data exploration and cleaning. The cleaned data was exported to SPSS version 25 for statistical analysis. Descriptive statistics was used to summarize categorical variables. Both bivariate and multivariable analyses were performed using logistic regression and adjusted odds ratios (AOR) with 95% confidence intervals for risk factors and maternal and neonatal complications associated with placenta praevia. *P* value < 0.05 was considered statistically significant.

### 2.11. Ethical Consideration

Ethical clearance for the proposed study was obtained from Addis Ababa University Institute of Review Board and Addis Ababa Health Bureau. Data was collected from patients' medical record, and confidentiality of the information was maintained throughout by excluding names as identification in the study.

## 3. Results

### 3.1. Sociodemographic Characteristics of the Cases and Controls

The number of deliveries that took place from September 2015 to January 2018 in both Gandhi Memorial and Tikur Anbessa Hospitals was 27195 and 17147, respectively, making the 44342 deliveries. Placenta praevia-complicated cases account for about 303 of all pregnancies, and hence, the magnitude was 0.7%. The mean age for case and control was 30.2 ± 5.769 and 30.24 ± 5.7 years, respectively. The mean length of hospital stay for cases was 14.27 ± 9.862 days which is significantly higher than controls (3.69 ± 2.25 days). Most of the women were at age of 25-29 years, residing in urban areas, and having their antenatal care visit during pregnancy (Table 1).

### 3.2. Antenatal Characteristics of Cases and Controls

With regard to parity, 158 (52.1%) of cases of placenta praevia were multiparous who gave birth to two or more neonates followed by primiparous75 (24.8%). Almost half of the cases (151 (49.9%)) tend to deliver preterm where 89 (29.4%) was early preterm and 62 (20.5%) late preterm. The percentage of cases that delivered at term was 152 (50.2%).

Caesarean section was the commonest mode of delivery, 285 (94.1%) among placenta praevia cases, and 75.5% of them had undergone regional anesthesia (spinal anesthesia) followed by general anesthesia 56 (18.5%) (Table 2).

### 3.3. Previous History of Cases and Controls

About 26.1% of placenta praevia cases had past history of caesarean section, which is higher when compared to controls (8.9%). Ninety-four (31%) had past history of abortion and 12 (4%) had previous molar pregnancy which was significantly higher than controls. Previous history of placenta praevia was 22 (7.3%), and women who had previous intrauterine fetal death were 9 (3%) (Table 3).

### 3.4. Maternal Complications of Cases and Controls

Women with placenta praevia who had developed postpartum hemorrhage and adherent placenta after delivery were 68 (22.4%) and 20 (6.6%), respectively. An insignificant number of cases had premature rupture of membranes (16 (5.3%)), surgical site infection (5 (1.7%)), hysterectomy (12 (4%)), and urinary tract infections (3 (0.5%)). Moderate anemia (91 (30%)) was commonest among the women with placenta praevia, and 29% of the cases had blood transfusion due to the large amount of blood losses (Table 4).

### 3.5. Neonatal Outcomes and Complications of Cases and Controls

The majority of neonates born to a placenta praevia case were male (198 (65.3%)), and only 6 (2%) of the neonates passed away due to the disease condition. Immediately after delivery, neonates were assessed of their Apgar score after 1 min and 5 min and were found to be <7 (71 (23.4%)) and <7 (6 (2%)), respectively, where significant numbers were at risk of asphyxia that necessitated resuscitation which was higher than women in control groups.

Birth weight was also evaluated where 166 (54.8%) was ≥2500 g, 132 (43.6%) was 1500-2499 g, and 5 (1.7) was <1500 g. Regarding the neonatal complications, respiratory distress syndrome (45 (14.9%)), intrauterine growth retardation (40 (13.2%)), hypothermia (23 (7.6%)), major congenital anomaly (spina bifida) (6 (2%)), neonatal hyperbilirubinemia (jaundice) (4 (1.3%)), and admission to the neonatal intensive care unit (75 (25.7%)) were the commonest complications. These findings were higher than in women of control groups (Table 5).

### 3.6. Risk Factors Associated with Placenta Praevia

Significant risk factors associated with placenta praevia after adjusting for potential confounder in multivariate logistic regression were high maternal age, multiparity, and women with history of caesarean section as shown in Table 6. Women with advanced maternal age (≥35) (AOR 6.3; 95% CI: 3.20, 12.51) have sixfold high risk to develop placenta praevia. Multiparity (AOR 2.2; 95% CI: 1.46, 3.46) and previous history of caesarean section (AOR 2.7; 95% CI: 1.64, 4.58) had an increased odds of placenta praevia. However, after adjusting for confounder, previous history of abortion, IUFD, and molar pregnancy had no significant association with placenta praevia (Table 7).

### 3.7. Maternal Complications Associated with Placenta Praevia

Table 8 depicts maternal complications associated with placenta praevia. After adjusting for confounders with a backward elimination model, postpartum hemoglobin < 12 g/dl and the need for blood transfusion 1-3 units were significantly associated with placenta praevia. Women with placenta praevia are fourteen times more likely to develop anemia (AOR 14.6; 95% CI: 6.48, 32.87) after delivery due to huge blood loss that necessitated blood transfusion 1-3 units (AOR 2.7; 95% CI: 1.10, 6.53) when compared to their counterparts. However, postpartum hemorrhage was not significantly associated after adjusting for confounder (Table 8).

### 3.8. Neonatal Complications Associated with Placenta Praevia

Table 6 depicts neonatal complications associated with placenta praevia. After adjusting for confounders with a backward elimination model, respiratory distress syndrome, intrauterine growth retardation (IUGR), and preterm birth were significantly associated with placenta praevia.

Neonates born to women with placenta praevia have fourfold increased risk of respiratory syndrome (AOR 4; 95% CI: 1.24, 13.85), sixfold increased risk of IUGR (AOR 6.3; 95% CI: 1.79, 22.38), and eightfold risk of preterm birth (AOR 8; 95% CI: 4.91, 12.90). Low birth weight, Apgar at l min < 7, hypothermia, and admission to NICU were not significantly associated with placenta praevia (Table 6).

## 4. Discussion

This study investigated the association between different risk factors and adverse maternal and neonatal outcomes with placenta praevia. Six neonatal deaths were recorded in this study. The magnitude of placenta praevia observed in this study was 7 in 1000 pregnancies. This was similar to study conducted in Siriraj Hospital in Thailand (0.7%) [17]. However, the magnitude was higher than the study conducted at Soroka University Medical Center, Israel, which was 0.38% [8]. The higher magnitude observed in this study has been attributed to increasing tendency to perform caesarean sections even without medical indication on maternal request.

Increased maternal age upsurges the risk of placenta praevia. This study found that advanced maternal age ≥ 35 connoted sixfold increase in risk of placenta praevia. This finding was similar to the retrospective case-control study conducted by Tuzovic and Ilijic. It states that advanced maternal age sixfold increases the risk of placenta praevia [5]. According to the study conducted by Choi et al., prevalence of placenta praevia increases as the maternal age advances [18]. This is thought to be due to atherosclerotic changes in the uterus resulting in underperfusion and infraction of the placenta, thereby increasing the size of the placenta.

Multiparity twofold increases the risk of placenta praevia. This result was in congruence with studies from Tuzovic and Ilijic and Kollmann et al. that reported women with parity of two or more showed an increased risk of placenta praevia. They showed a greater risk of placenta praevia with higher parity [5, 19]. This may be due to endometrial scarring at the site of prior placental attachments resulting in lower placental implantation, and another possibility may be due to atherosclerotic changes of blood vessels, which lead to decreased uteroplacental blood flow, which in turn leads to large placenta encroaching on the cervical os with repeated pregnancies.

According to this study, patients who had previous delivery by caesarean section have about three times increased risk of placenta praevia. Most studies have reported an association between previous caesarean section and placenta praevia. Similarly, In a meta-analysis of 170640 pregnant women, a pattern of risk factors for placenta praevia was found with the increasing number of caesarean section deliveries [8].

Another cohort study in United Kingdom NHS hospital also shows that among 131,731 women who had elective CS, 4332 women had placenta praevia at term making 32.9 per 1000 elective CS [13].

This is because surgical disruption of the uterine cavity is known to cause lasting damage to the myometrium and endometrium. If a previous caesarean section is performed, there is a problem of angiogenesis in the previous operation site that may cause partial hypoxia. This hypoxia leads to incomplete decidualization and abnormal trophoblast invasion that can cause placental adhesion [20].

This study found that although a significant number of women had previous history of abortion, the known risk factor for placenta praevia was not significantly associated after being adjusted for confounders in multivariate regression. It might be due to the study design and sample size. This was in contrast with the finding of Tuzovic and Ilijic that the percentage of previous abortions was significantly higher among women with placenta praevia, which yielded a risk of 2.75. The mechanism of how previous abortions predispose to placenta praevia development could be explained with possible endometrial damage during repeated abortions, which impedes successful fundal implantation of the placenta [5].

Massive hemorrhage either antepartum, intrapartum, or postpartum associated with placenta praevia may lead to decline in the maternal hemoglobin level. According to this study, women with placenta praevia were fourteen times more likely to have a decreased postpartum hemoglobin level less than 12 g/dl that led them to postpartum anemia. This result was comparable with previous study done by Sheiner where women with placenta praevia were about six times more likely to have postpartum anemia [8].

The amount of blood transfused was significantly more in women with major placenta praevia. This denotes that the increased blood transfusion is due to the increased bleeding caused by placenta praevia. According to this study, women with placenta praevia necessitated about threefold blood transfusion. The finding was similar to the retrospective study conducted at the obstetric unit of Abha General Hospital, Saudi Arabia; the odds of blood transfusion > 3 units in major placenta praevia were threefold higher than their counterparts [21].

A large number of patients (*N* = 166) had postpartum hemorrhage associated with placenta praevia. However, there was no significant association between postpartum hemorrhage and placenta praevia after adjusting for confounders in multiple logistic regressions. In contrast to this finding, previous studies showed that placenta praevia increases the risk of postpartum hemorrhage. This might be due to the sample size difference. This is explained by the implantation of placenta in a previous scar, which may go deep preventing placental separation. This may provoke severe hemorrhage during and after delivery because the lower segment does not constrict well the maternal blood supply [13–16].

Placenta praevia is linked to a number of adverse neonatal outcomes including preterm delivery. In this study, neonates born to women with placenta praevia were eight times more likely to be born preterm. This result was comparable to the Danish national cohort study on the neonatal outcome in singleton pregnancies with placenta praevia from 2001 to 2006; neonates born after pregnancies with placenta praevia had about ninefold risk of being born at a gestational age below 37 weeks [22].

Neonates born in early gestational age (preterm) due to placenta praevia were at risk of respiratory distress syndrome. This study found that neonates were eight times more likely to develop respiratory distress syndrome. This finding was consistent with the study conducted by Crane et al., where neonates born to placenta praevia women have fivefold increased risk of developing respiratory distress syndrome [23]. We speculate that placenta praevia was not directly contributing to respiratory distress syndrome, but through other associated risk factors for respiratory distress syndrome.

According to this finding, placenta praevia connoted sixfold risk of developing intrauterine growth restriction in neonates. Similarly, the study conducted in Lady Reading Hospital, Pakistan, showed that neonates born to placenta praevia mothers have developed intrauterine growth restriction [24].

One hundred sixty-six cases of neonates born to placenta praevia mothers have low birth weight. However, there is no significant association between placenta praevia and low birth weight of neonate after adjusting for confounders. In contrast, several previous studies found association between them. These discrepancies might be due to difference in study design and sample size [12, 25].

### 4.1. Strength and Limitation of the Study

The study included all placenta praevia cases in the given study time frame and place and thus minimize selection bias. Controls were selected to minimize possible confounders that affect the results. However, the study had limitation that it was difficult to get full information about the patient. It was a hospital-based study, so its results may not be applicable on the whole population of Ethiopian pregnant women.

## 5. Conclusion and Recommendation

This study showed that the magnitude of placenta praevia was 7 in 1000 pregnancies. Advanced maternal age, multiparity, and previous histories of caesarean section were significantly associated risk factors of placenta praevia. Adverse maternal outcomes associated with placenta praevia were postpartum anemia and the need for blood transfusion after significant amount blood loss due to the disease condition and its complications. Neonates born to women with placenta praevia were also at risk of being born preterm, intrauterine growth restriction, and respiratory distress syndrome.

Patients with placenta praevia should be considered as high risk, and compatible blood should always be available for such cases before considering caesarian section. Family planning should also be emphasized as a strategy towards reduction of parity, caesarean section rate, and thereby the incidence of placenta praevia. Strategies and protocols should be settled to reduce the rate of caesarean section, and senior staffs have to be involved in the management of cases of placenta praevia.

## Figures and Tables

**Table 1:** Sociodemographic characteristics of the cases and controls in Tikur Anbessa Specialized Referral and Gandhi Memorial Hospitals, Ethiopia, 2018.

Sociodemographic characteristics		Case *N* = 303	Control *N* = 303	Total *N* = 606
	
Age	<25	41 (33.3%)	101 (13.5%)	142 (23.4%)
	25-29	105 (37.6%)	114 (34.7%)	219 (36.1%)
	30-35	85 (23.4%)	71 (28.1%)	156 (25.7%)
	>35	72 (5.6%)	17 (23.8%)	89 (14.7%)

Residence	Addis Ababa	275 (93.7%)	284 (90.8%)	559 (92.2%)
	Oromia	26 (5.9%)	18 (8.6%)	44 (7.3%)
	Amhara	2 (0.3%)	1 (0.7%)	3 (0.5%)

Antenatal follow-up	Yes	291 (96.0%)	263 (86.8%)	554 (91.4%)
	No	12 (4%)	40 (13.2%)	52 (8.6%)

**Table 2:** Antenatal characteristics of case and controls in Tikur Anbessa Specialized Referral and Gandhi Memorial Hospitals, Ethiopia, 2018.

Antenatal characteristics		Case (*n* = 303)	Control (*n* = 303)	Total (*n* = 606)
Parity	Nulliparous	70 (23.1%)	99 (32.7%)	169 (27.9%)
	Primiparous	75 (24.8%)	124 (40.9%)	199 (32.8%)
	Multiparous	158 (52.1%)	80 (26.4%)	238 (39.3%)

Gestational age at delivery	28-33 weeks	89 (29.4%)	0 (0.0%)	89 (14.7%)
	34-36 weeks	62 (20.5%)	25 (8.3%)	87 (14.4%)
	≥37 weeks	152 (50.2%)	278 (91.7)	430 (71%)

Mode of delivery	C/S	285 (94.1%)	79 (26.1%)	364 (60.1%)
	SVD	18 (5.9%)	197 (65%)	215 (35.5%)
	Instrumental	0 (0%)	27 (8.9%)	27 (4.5%)

Types of anesthesia	General	56 (18.5%)	8 (2.6%)	64 (10.6%)
	Spinal	229 (75.5%)	71 (23.4%)	314 (51.8%)
	No anesthesia	18 (5.9%)	224 (73.9%)	228 (37.6%)

**Table 3:** Previous history of cases and controls in Tikur Anbessa Specialized Referral and Gandhi Memorial Hospitals, Ethiopia, 2018.

Previous history		Case (*n* = 303)	Control (*n* = 303)	Total (*n* = 606)
C/S	Yes	79 (26.1%)	27 (8.9%)	106 (17.5%)
	No	224 (73.9%)	276 (91.1%)	500 (82.5%)
Abortion	Yes	94 (31.0%)	56 (18.5%)	150 (24.8%)
	No	209 (69%)	247 (81.5%)	456 (75.2%)
Molar pregnancy	Yes	12 (4%)	5 (1.7%)	17 (2.8%)
	No	291 (96%)	298 (98.3%)	589 (97.2%)
Placenta praevia	Yes	22 (7.3%)	0 (0%)	22 (3.6%)
	No	281 (92.7%)	303 (100%)	584 (96.4%)
IUFD	Yes	9 (3%)	5 (1.7%)	14 (2.3%)
	No	294 (97%)	298 (98.3%)	592 (97.7%)

**Table 4:** Maternal complications of cases and controls in Tikur Anbessa Specialized Referral and Gandhi Memorial Hospitals, Ethiopia, 2018.

Maternal complications		Case (*n* = 303)	Control (*n* = 303)	Total (*n* = 606)
PPH	Yes	68 (22.4%)	7 (2.3%)	75 (12.4%)
	No	235 (77.6%)	296 (97.7%)	531 (87.6%)

Adherent placenta	Yes	20 (6.6%)	0 (0%)	20 (3.3%)
	No	283 (93.4%)	303 (100%)	586 (96.7%)

Surgical site infection	Yes	5 (1.7%)	5 (1.7%)	10 (1.7%)
	No	298 (98.3%)	298 (98.3%)	596 (98.3%)

PROM	Yes	16 (5.3%)	16 (5.3%)	32 (5.3%)
	No	287 (94.7%)	287 (94.7%)	574 (94.7%)

Hysterectomy	Yes	12 (4%)	0 (0%)	12 (2%)
	No	291 (96%)	303 (100%)	594 (98%)

UTI	Yes	3 (0.5%)	0 (0%)	3 (1%)
	No	300 (50%)	303 (50)%	603 (100%)

Anemia	Severe anemia	3 (1%)	0 (0%)	3 (0.5%)
	Moderate anemia	91 (30%)	7 (2.3%)	98 (16.2%)
	No anemia	209 (69%)	296 (97.7%)	505 (83.3%)

Blood transfusion	1-3 units	104 (34.3%)	14 (4.6%)	118 (19.5%)
	≥4	18 (5.9%)	0 (0%)	18 (3%)
	No transfusion	181 (59.7%)	289 (95.4%)	470 (77.6%)

Hospital stay	<14 days	188 (62%)	303 (100%)	491 (81%)
	≥14 days	115 (38%)	0 (0%)	11519.0%

**Table 5:** Neonatal outcome and complications of cases and controls in Tikur Anbessa Specialized Referral and Gandhi Memorial Hospitals, Ethiopia, 2018.

Neonatal outcome and complications		Case (*n* = 303)	Control (*n* = 303)	Total (*n* = 606)
Sex	Male	198 (65.3%)	190 (62.7%)	388 (64%)
	Female	105 (34.7%)	113 (37.3%)	218 (36%)

Birth outcome	Alive	297 (98%)	302 (99.7%)	599 (98.8%)
	Dead	6 (2%)	1 (0.3%)	7 (1.2%)

APGAR score at 1 min	<7	71 (23.4%)	22 (7.3%)	93 (15.3%)
	7-10	232 (76.6%)	281 (92.7%)	513 (84.7%)

APGAR score at 5 min	<7	6 (2%)	0 (0%)	6 (1%)
	7-10	297 (98%)	303 (100%)	600 (99%)

Birth weight	<1000	2 (0.7%)	4 (1.3%)	6 (1%)
	1000-1499	3 (1%)	10 (3.3%)	13 (2.1%)
	1500-2499	132 (43.6%)	57 (18.8%)	189 (31.2%)
	≥2500	166 (54.8%)	232 (76.6%)	398 (65.7%)

Respiratory distress syndrome	Yes	45 (14.9%)	4 (1.3%)	49 (8.1%)
	No	258 (85.1%)	299 (98.7%)	557 (91.9%)

Spina bifida	Yes	6 (2%)	3 (1%)	9 (1.5%)
	No	297 (98%)	300 (99%)	597 (98.5%)

IUGR	Yes	40 (13.2%)	3 (1%)	43 (7.1%)
	No	263 (86.8%)	300 (99%)	563 (92.9%)

Neonatal jaundice	Yes	4 (1.3%)	3 (1%)	7 (1.2%)
	No	299 (98.7%)	300 (99%)	599 (98.8%)

Hypothermia	Yes	23 (7.6%)	2 (0.7%)	25 (4.1%)
	No	280 (92.4%)	301 (99.3%)	581 (95.9%)

Admission to NICU	Yes	78 (25.7%)	12 (4%)	90 (14.9%)
	No	225 (74.3%)	291 (96%)	516 (85.1%)

**Table 6:** Binary and multivariate logistic regression for neonatal complications associated with placenta praevia in Tikur Anbessa Specialized Referral and Gandhi Memorial Hospitals, Ethiopia, 2018.

Neonatal complications		Case *N* (303)	Control *N* (303)	COR (95% CI)	AOR (95% CI)
Low birth weight	Yes	166	232	.37 (.26, .53)	1.2 (.77, 1.96)
	No	137	71	1.0	1.0

APGAR score at 1 min < 7	Yes	20	4	4 (2.35, 6.50)^∗∗^	1.2 (.61, 2.45)
	No	283	299	1.0	1.0

Respiratory distress syndrome	Yes	45	4	13 (4.63, 36.74)^∗∗^	4 (1.24, 13.85)^∗^
	No	258	299	1.0	1.0

Hypothermia	Yes	23	2	12.4 (2.89, 52.92)^∗^	4.451 (.843, 23.50)
	No	280	301	1.0	1.0

IUGR	Yes	40	3	15.2 (4.65, 49.74)^∗∗^	6.3 (1.79, 22.38)^∗^
	No	263	300	1.0	1.0

Admission to NICU	Yes	78	12	8.4 (4.47, 15.82)^∗∗^	.52 (.14, 1.96)
	No	225	291	1.0	1.0

Preterm birth	Yes	151	25	11 (6.92, 17.62)^∗∗^	8 (4.91, 12.90)^∗∗^
	No	152	278	1.0	1.0

COR: crude odds ratio; AOR: adjusted odds ratio; CI: confidence interval. ^∗^Statistically significant variables at *P* < 0.05. ^∗∗^Statistically significant variables at *P* < 0.001. Hosmer‐Lemeshow goodness‐of‐fit = 0.324.

**Table 7:** Binary and multivariate logistic regression of risk factors associated with placenta praevia in Tikur Anbessa Specialized Referral and Gandhi Memorial Hospitals, Ethiopia, 2018.

Risk factors		Case *N* (303)	Control *N* (303)	COR (95% CI)	AOR (95% CI)
Maternal age	15-24	41	101	1.0	1.0
	25-34	190	185	2.5 (1.67, 3.38)^∗∗^	2 (1.26, 3.04)^∗^
	≥35	72	17	10.4 (5.45, 19.80)^∗∗^	6.3 (3.20, 12.51)^∗∗^

Parity	Nulliparous	70	99	1.2 (.77, 1.78)	1.5 (.98, 2.40)
	Primiparous	75	124	1.0	1.0
	Multiparous	158	80	3.2 (2.20, 4.84)^∗∗^	2.2 (1.46, 3.46)^∗∗^

Previous history of C/S	Yes	79	27	3.6 (2.25, 5.77)^∗∗^	2.7 (1.64, 4.58)^∗∗^
	No	224	276	1.0	1.0

Previous history of abortion	Yes	94	56	2 (1.36, 2.90)^∗^	1.5 (.98, 2.25)
	No	209	247	1.0	1.0

Previous molar pregnancy	Yes	12	5	2.5 (.85, 7.06)^∗^	2.8 (.92, 8.49)
	No	291	298	1.0	1.0

Previous IUFD	Yes	9	5	1.8 (.604, 5.51)^∗^	1.2 (.35, 4.02)
	No	294	298	1.0	1.0

COR: crude odds ratio; AOR: adjusted odds ratio; CI: confidence interval. ^∗^Statistically significant variables at *P* < 0.05. ^∗∗^Statistically significant variables at *P* < 0.001. Hosmer‐Lemeshow goodness‐of‐fit = 0.63.

**Table 8:** Binary and multivariate logistic regression for maternal complications associated with placenta praevia in Tikur Anbessa Specialized Referral and Gandhi Memorial Hospitals, Ethiopia, 2018.

Maternal complications		Case *N* (303)	Control *N* (303)	COR (95% CI)	AOR (95% CI)

Postpartum	Yes	94	7	19 (8.65, 41.82)^∗∗^	14.6 (6.48, 32.87)^∗∗^
Hemoglobin < 12 g/dl	No	209	296	1.0	1.0
Blood transfusion 1-3 units	Yes	104	14	12 (6.58, 21.34)^∗∗^	2.7 (1.10, 6.53)^∗^
	No	181	289	1.0	1.0
PPH	Yes	68	7	12 (5.52, 27.14)^∗∗^	2.2 (.71, 6.57)
	No	235	296	1.0	1.0

COR: crude odds ratio; AOR: adjusted odds ratio; CI: confidence interval. ^∗^Statistically significant variables at *P* < 0.05. ^∗∗^Statistically significant variables at *P* < 0.001. Hosmer‐Lemeshow goodness‐of‐fit = 0.75.

## Data Availability

The data used to support the findings of this study are available from the corresponding author upon request.

## Abbreviations

COR: Crude odds ratio
AOR: Adjusted odds ratio
CI: Confidence interval.
